# Supplementary material for: R2‐P2 rapid‐robotic phosphoproteomics enables multidimensional cell signaling studies
Source: Mol Syst Biol. 2019 Dec 19;15(12):e9021. doi: 10.15252/msb.20199021 (PMC6920700; doi:10.15252/msb.20199021)
Supplement: Supplementary file 1 — Appendix [file MSB-15-e9021-s001.pdf]

Standard Operation Procedure: Automated phosphoproteomic sample preparation with R2-P2

1 Document Information 2
  1.1 Purpose 2
  1.2 Scope 2
  1.3 Outline 2
  1.4 Abbreviations 2
2 General Suggestions 2
3 R2-P1 3
  3.1 Reagent and Material List 3
  3.2 Protocol 4
    3.2.1 Important Notes 4
    3.2.2 Experiment Planning 4
    3.2.3 Carboxylated Magnetic Bead Preparation 4
    3.2.4 96-well plate preparation for R2-P1 5
    3.2.5 R2-P1 KingFisher™ Flex protocol 6
4 R2-P2 10
  4.1 Reagent and Material List 10
  4.2 Protocol 10
    4.2.1 Important Notes 10
    4.2.2 Experiment Planning 10
    4.2.3 Magnetic Fe^{3+}-IMAC Bead Preparation and Recycling 11
    4.2.4 Fe^{3+}-IMAC plate preparation for R2-P2 11
    4.2.5 R2-P2 KingFisher™ Flex protocol 13

1 Document Information

1.1 Purpose

This standard operating protocol (SOP) document describes rapid-robotic-proteomics (R2-P1) and rapid-robotic-phosphoproteomics (R2-P2) protocols for automated sample processing to prepare proteomic and phosphoproteomics samples in a 96-well format for LC-MS analysis. R2-P2 is a combination of solid-phase-enhanced sample preparation using carboxylated magnetic beads and phosphopeptide enrichment with Fe^{3+}-IMAC, Ti^{4+}-IMAC, TiO_2 or Zr^{4+}-IMAC magnetic particles using a KingFisher™ Flex magnetic bead processor. We have extensively tested the method and provide a detailed protocol for the manual and automated steps.

1.2 Scope

The provided information will allow investigators to implement R2-P2 as a standard sample processing method for proteomic workflows resulting in MS-ready peptide samples for total proteome and/or phosphoproteome analysis.

1.3 Outline

The document is organized in the following sections:

General suggestions: Considerations for planning experiments with R2-P2.

Protocol for R2-P1: A validated protocol for automated solid-phase-enhanced sample preparation in a 96-well plate format by a KingFisher™ Flex magnetic handling robot. Starts from cell or tissue lysates and ends with peptide samples ready for total proteome analysis by LC-MS and for phosphopeptide enrichments.

Protocol for R2-P2: Combination of R2-P1 and automated phosphopeptide enrichment carried out in a 96-well plate format by a KingFisher™ Flex magnetic handling robot. Starts with desalted peptides and ends with samples ready for phosphoproteome analysis by LC-MS.

1.4 Abbreviations

R2-P1 – rapid-robotic-proteomics
R2-P2 – rapid-robotic-phosphoproteomics
ACN – Acetonitrile
AmBic – Ammonium bicarbonate
EtOH – Ethanol
IMAC – Immobilized-metal affinity chromatography
LC – Liquid chromatography
MS – Mass Spectrometry
MeOH – Methanol
TFA - Trifluoroacetic Acid

2 General Suggestions

Protease inhibitors: We have seen that Pierce protease inhibitors (#A32963) are not efficiently removed by R2-P1 and lead to decreased digestion efficiency by trypsin or LysC if they are present at >0.2x in the digestion buffer. To circumvent this, one of the following strategies, or combination of them, can be chosen:

- To use a denaturing buffer (e.g. 8 M urea) without protease inhibitors for lysis

- To produce cell lysates at protein concentration higher than 4 mg/ml
- To dilute the lysate 4-fold prior to digestion

Phosphatase inhibitors: We have seen that a common phosphatase inhibitor mix (50 mM sodium fluoride, 10 mM sodium pyrophosphate, 50 mM sodium beta-glycerophosphate, 1 mM sodium orthovanadate) is not efficiently removed by R2-P1, leading to competition during phosphopeptide enrichments. We could not observe any negative effect of phosphatase inhibitors on LC-MS analysis for total proteome analysis. We recommend using denaturing buffers (e.g. 8 M urea) without phosphatase inhibitors for lysis.

Precipitates: Depending on the sample, we have observed insoluble particles due to precipitation at a few steps in the protocol that we indicate below. It is critical that phosphopeptide enrichment is performed on a clarified peptide mixture for maximal efficiency. If precipitation is observed at any step after protein digestion the plate needs to be centrifuged for 10 minutes at maximal speed and supernatant transferred to a new plate. If this does not help to remove visible precipitates longer centrifugation is needed. Alternatively, samples can be transferred to PCR tube strips and centrifuged in a PCR tube centrifuge at higher speeds.

Magnetic particles for phosphopeptide enrichment: The phosphopeptide enrichment described in this protocol is performed using Fe^{3+}-IMAC magnetic particles. In our hands, Fe^{3+}-IMAC is the most efficient material for phosphopeptide enrichment; it is easy to use and cheap (since the Fe^{3+} can be stripped and reloaded multiple times). However, we have also successfully tested R2-P2 with Ti^{4+}-IMAC, TiO_2 and Zr^{4+}-IMAC microspheres from MagReSyn following the provided instructions. The results were of comparable quality and different resins provided orthogonal phosphopeptide identification.

3 R2-P1

3.1 Reagent and Material List

Item	Vendor	Catalog #
KingFisher™ Flex	Thermo Scientific	
KingFisher 96 KF microplate (200µL)	Fisher Scientific	22-387-030
KingFisher 96 microtiter DW plate	Fisher Scientific	22-387-032
KingFisher 96 tip comb for DW magnets	Fisher Scientific	22-387-029
SpeedVac Vacuum Concentrator	various	
Plate or PCR tube centrifuge	various	
Sera-Mag SpeedBead Carboxylate-Modified Magnetic Particles (Hydrophilic)	GE Life Sciences	45152105050250
Sera-Mag SpeedBead Carboxylate-Modified Magnetic Particles (Hydrophobic)	GE Life Sciences	65152105050250
Water LC-MS grade	Fisher Scientific	7732-18-5
Acetonitrile LC-MS grade	Fisher Scientific	75-05-8
Formic acid LC-MS grade	Fisher Scientific	A117-50
Ethanol 200 Proof (100%)	Decon Labs	2701
Ammonium bicarbonate	Sigma-Aldrich	A6141
Sequencing grade modified trypsin	Promega	V5111
Methanol LC-MS grade (optional)	Fisher Scientific	A456-4
Acetic acid glacial HPLC grade (optional)	Fisher Scientific	A35-500
C8 Extraction Disks 3M™ Empore™ (optional)	Fisher Scientific	14-386

3.2 Protocol

3.2.1 Important Notes

- All steps are performed at room temperature, unless stated otherwise.
- The following protocol is for 250 µg of protein digest in one well. The protocol has been successfully tested for protein input amounts from 25 µg to 500 µg.
- The protocol has been extensively tested with lysates performed in urea buffer (8 M urea, 150 mM NaCl, 100 mM Tris pH 8), however other lysis buffers should work as well.
- With the recommended buffer volumes and ratios, the maximal amount of protein that can be processed in one well is 500 µg.
- We perform digests for 3.5 hours at 37 ºC, however these parameters can easily be adjusted as needed in the protocol.
- All plate pipetting steps are performed with 8- or 12- multi-channel pipettes.

3.2.2 Experiment Planning

1. Determine the amount of protein sample that needs to be processed, considering:
    a. For proteome analysis only, we recommend starting with 25 µg of protein.
    b. For phosphoproteomic analysis we recommend starting with a minimum of 100 µg protein, with an optimal starting amount of 200 µg – 500 µg. This is sample dependent and needs to be tested.
2. Determine the protein concentration of clarified cell lysate(s) by BCA assay or equivalent. Reduce and alkylate cysteines by preferred method. Adjust protein concentration in samples to 1 µg/µl with lysis buffer.
3. Determine the amount of magnetic carboxylated beads to be used. We recommend using 1 µl of 10 µg/µl of carboxylated bead mix per µg of protein to be processed.
4. Determine the volumes of the solutions to be used per well in the different plates:
    a. Binding plate:
        i. Lysate volume (V_{Lysate}) = Calculate lysate volume to reach desired protein amount, keeping a protein concentration of 1 µg/µl.
        ii. 100% EtOH volume = V_{Lysate} (to reach 50% EtOH v/v)
    b. Wash plates (3x): 80% EtOH volume = 2 x V_{Lysate}
    c. Elution 1 plate:
        i. 150 µl 25 mM AmBic pH 8.2
        ii. Trypsin or LysC: 1 µg enzyme : 100 µg protein
    d. Elution 2 plate: 100 µl water
5. Determine the type of plates to be used for the different solutions: For volumes of 50 µl – 150 µl per well use Kingfisher microplates (shallow well) and for 150 µl – 1000 µl per well use Kingfisher microtiter DW plates (deep well).

3.2.3 Carboxylated Magnetic Bead Preparation

1. Take both (hydrophilic and hydrophobic) magnetic carboxylated bead stocks off the fridge, warm to room temperature and vortex gently to fully suspend magnetic beads.
2. Beads come at a stock concentration of 50 µg/µl. Take the required amount of beads and mix hydrophilic and hydrophobic beads at a 1:1 ratio. Dilute to a total bead concentration of 1 µg/µl, wash the beads three times with water keeping the beads at 1 µg/µl on a magnetic rack in an Eppendorf or Falcon tube and resuspend beads in water at the working concentration of 10 µg/µl.

3.2.4 96-well plate preparation for R2-P1

1. Prepare an empty plate containing the tip comb.
2. Prepare the Bead plate by adding the calculated amount of beads to the plate. If the volume is < 50 µl, fill up to 50 µl with water.
3. Transfer the calculated volumes of cell lysate (1 µg/µl) to the Binding plate.
4. Add 100% EtOH to the Binding plate to reach 50% EtOH (v/v) (no mixing or pipetting required).
5. Prepare the 3 Wash plates by dispensing 80% EtOH.
6. Prepare Elution-2 plate by dispensing water.
7. Start the R2-P1 Kingfisher program and follow the instructions on the robot. Leave the space for Elution-1 plate empty and start the protocol.
8. Start preparing Elution-1 plate on ice 25 min into the protocol.
9. The R2-P1 Kingfisher program will pause after approximately 35 minutes. Follow the instructions of the robot, load Elution-1 plate and resume the program.
10. Remove all plates after the program finishes.
11. Transfer solution in Elution-2 plate to Elution-1 plate.
12. Stop the enzymatic digestion by adding formic acid to the digest to pH < 2 (usually final formic acid concentration is 3-5%).
13. Depending on sample, varying degrees of precipitations might be observed after digestion. For efficient downstream sample processing (e.g. phosphopeptide enrichment or LC-MS analysis) remove precipitates by centrifugation and transfer the supernatant to a new plate.
14. Take a sample aliquot for total proteome analysis from supernatant, see optional step 15 or transfer to a MS vial or MS sample plate.
15. (Optional step that we recommended when processing many samples in parallel): To avoid any carryover of magnetic beads to the LC system, filter samples through a C8 stage tip or stage tip plate. For this, pack a stage tip with one layer of C8 material and perform the following steps:
    a. Condition with 30 µl MeOH.
    b. Wash with 30 µl 100% ACN.
    c. Wash with 30 µl 70% ACN, 0.25% acetic acid.
    d. Hold stage tip on top of MS sample vial.
    e. Adjust sample to 50% ACN and pass sample through stage tip, collecting eluate in the MS vial.
    f. Elute with 30 µl 70% ACN, 0.25% acetic acid, collecting this second eluate also in the MS vial.

16. Dry samples down in a speedvac. Dried samples can be store at -20 ºC. Resuspend in 4% formic acid and 3% ACN prior to LC-MS analysis.
17. The rest of the plate is dried down and can be stored at -20 ºC for subsequent phosphopeptide enrichment.

3.2.5 R2-P1 KingFisher™ Flex protocol

General info

Protocol information

Protocol name	R2-P1_2019
Modified by	admin
Kit name	R2-P1
Description	R2-P1 KingFisher Flex 96 protocol for desalting and digestion of 250 ug protein.<br>Mario Leutert, Villen Laboratory, University of Washington

Reagent info

Beads		Microtiter DW 96 plate	
Name	Well volume [µl]	Total reagent volume [µl]	Type
Carboxyl. Beads 10ug/ul (1ul Beads/1ul)	250	-	Reagent

Binding		Microtiter DW 96 plate	
Name	Well volume [µl]	Total reagent volume [µl]	Type
100% EtOH	250	-	Sample
Lysate (1ug/ul protein)	250	-	Reagent

Wash 1		Microtiter DW 96 plate	
Name	Well volume [µl]	Total reagent volume [µl]	Type
80% EtOH	500	-	Reagent

Wash 2		Microtiter DW 96 plate	
Name	Well volume [µl]	Total reagent volume [µl]	Type
80% EtOH	500	-	Reagent

Wash 3		Microtiter DW 96 plate	
Name	Well volume [µl]	Total reagent volume [µl]	Type
80% EtOH	500	-	Reagent

Tip Storage Plate		KingFisher 96 KF plate	
Name	Well volume [µl]	Total reagent volume [µl]	Type
-	-	-	-

Elution 1 - Trypsin		KingFisher 96 KF plate	
Name	Well volume [µl]	Total reagent volume [µl]	Type
25mM Ambic, Trypsin 1:100	100	-	Reagent

Elution 2		KingFisher 96 KF plate	
Name	Well volume [µl]	Total reagent volume [µl]	Type
H2O	100	-	Reagent

Steps data

Tip1	96 DW tip comb	
Pick-Up	Tip Storage Plate	
Mix and collect Beads	Beads	
Beginning of step	Precollect	No
	Release beads	No
Mixing / heating:	Mixing time, speed	00:00:30, Bottom mix
	Heating during mixing	No
End of step	Postmix	No
	Collect count	5
	Collect time [s]	30
Capture Proteins	Binding	
Beginning of step	Precollect	No
	Release time, speed	00:01:00, Bottom mix
Mixing / heating:	Shake 1 time, speed	00:12:00, Medium
	Shake 2 time, speed	00:01:00, Slow
	Heating during mixing	No
End of step	Postmix	No
	Collect count	5
	Collect time [s]	30
Wash 1	Wash 1	
Beginning of step	Precollect	No
	Release time, speed	00:01:00, Medium
Mixing / heating:	Mixing time, speed	00:02:00, Medium
	Heating during mixing	No
End of step	Postmix	No
	Collect count	5
	Collect time [s]	30
Wash2	Wash 2	
Beginning of step	Precollect	No
	Release time, speed	00:01:00, Medium
Mixing / heating:	Mixing time, speed	00:02:00, Medium
	Heating during mixing	No
End of step	Postmix	No
	Collect count	5
	Collect time [s]	30
Wash3	Wash 3	
Beginning of step	Precollect	No
	Release time, speed	00:01:00, Medium
Mixing / heating:	Mixing time, speed	00:02:00, Medium
	Heating during mixing	No
End of step	Postmix	No
	Collect beads	No

Pause - Load Elution1		Elution 1 - Trypsin	
		Message	Insert Elution1 - Trypsin
		Dispensing volume [µl]	0
Pause - Load Elution2		Elution 2	
		Message	Insert Elution2
		Dispensing volume [µl]	0
Collect Beads Wash 3		Wash 3	
		Collect count	5
		Collect time [s]	30
Protein Digestion and Elution		Elution 1 - Trypsin	
	Beginning of step	Precollect	No
		Release time, speed	00:01:00, Bottom mix
	Mixing / heating:	Shake 1 time, speed	01:44:00, Slow
		Shake 2 time, speed	00:01:00, Medium
		Shake 3 time, speed	01:45:00, Slow
		Heating temperature [°C]	37
		Preheat	No
	End of step	Postmix	No
		Collect count	5
		Collect time [s]	30
Elution 2		Elution 2	
	Beginning of step	Precollect	No
		Release time, speed	00:01:00, Bottom mix
	Mixing / heating:	Shake 1 time, speed	00:01:00, Bottom mix
		Shake 2 time, speed	00:04:00, Medium
		Heating during mixing	No
	End of step	Postmix	No
		Collect count	5
		Collect time [s]	30
Dispose Beads		Beads	
		Release time, speed	00:00:30, Bottom mix
Leave		Tip Storage Plate	

4 R2-P2

4.1 Reagent and Material List

Item	Vendor	Catalog #
KingFisher™ Flex	Thermo Scientific	
KingFisher 96 KF microplate (200µL)	Fisher Scientific	22-387-030
KingFisher 96 tip comb for DW magnets	Fisher Scientific	22-387-029
SpeedVac Vacuum Concentrator	various	
Plate or PCR tube centrifuge	various	
Bath sonicator	various	
Fe-NTA MagBeads	Cube Biotech	31501-Fe
Water LC-MS grade	Fisher Scientific	7732-18-5
Acetonitrile LC-MS grade	Fisher Scientific	75-05-8
Formic acid LC-MS grade	Fisher Scientific	A117-50
Trifluoroacetic acid LC-MS grade	Fisher Scientific	A116-50
Ammonia (ammonium hydroxide), NH_4OH	Sigma-Aldrich	221228-A
Ferric chloride, FeCl3 (optional)	Sigma-Aldrich	F2877
EDTA (optional)	Sigma-Aldrich	EDS
Methanol LC-MS grade (optional)	Fisher Scientific	A456-4
Acetic acid glacial HPLC grade (optional)	Fisher Scientific	A35-500
C8 Extraction Disks 3M™ Empore™ (optional)	Fisher Scientific	14-386

4.2 Protocol

4.2.1 Important Notes

- Any precipitate present in the peptide sample significantly reduces the selectivity of phosphopeptide enrichment and needs to be removed by centrifugation prior to the binding step.
- Do not leave magnetic beads without liquid for longer than 1 min.
- Do not leave magnetic beads in aqueous solutions for longer than 2h.
- If magnetic beads are clumped or aggregated, sonicate them briefly in a bath sonicator.
- Magnetic Fe^{3+}-IMAC beads can be reused by stripping the ion metal and reloading it. We have observed no change in performance after reuse for up to 1 year.
- All plate pipetting steps are performed with 8- or 12- multi channel pipettes.

4.2.2 Experiment Planning

1. Determine the amount of magnetic Fe^{3+}-IMAC beads to be used. We recommend using 2.5 µL of 5% beads per 10 µg of peptides. However, this is sample dependent and might need to be adjusted.
2. Determine the volumes of the solutions to be used per well in the different plates:
    a. Binding plate: 150 µl 80%ACN, 0.1%TFA
    b. Wash plates (3x): 150 µl 80%ACN, 0.1%TFA
    c. Elution plate: 50 µl 50% ACN, 2.5% NH_4OH
    d. Neutralization solution for elution plate: 30 µl 75% ACN, 10% formic acid

4.2.3 Magnetic Fe^{3+}-IMAC Bead Preparation and Recycling

Magnetic bead preparation steps are performed on a magnetic rack in 2ml tubes. For washing, remove tubes from the rack, homogenize beads by flicking the tube, and remove all liquid.

Usage of new Fe^{3+}-NTA MagBeads:

1. PureCube Fe-NTA MagBeads are delivered as a 25% suspension and are ready to use for phosphopeptide enrichment.
2. Before use, dilute beads to 5% and wash three times with 80% ACN, 0.1% TFA. For all further handling steps beads are kept at 1 ml aliquots at a working concentration of 5%.

Storage of used Fe^{3+}-NTA MagBeads:

1. After R2-P2, beads can be recollected before they dry out.
2. Wash once with 1 ml 50% ACN, 50% MeOH, 0.01% acetic acid.
3. Store beads in 1 ml of the same buffer at 4 ºC until further use.

Stripping and reloading of Fe^{3+}-NTA MagBeads:

1. Wash beads three times with 1 ml of water.
2. Wash once with 1 ml 40-100 mM EDTA, pH 8.
3. Resuspend beads in 1 ml 40-100 mM EDTA, pH 8 and incubate for 30 minutes while shaking or rotating the tubes. Ensure that the beads remain in solution.
4. Wash beads three times with 1 ml of water.
5. Wash once with 1 ml 10 mM $FeCl_3$.
6. Resuspend in 1 ml 10 mM $FeCl_3$ and incubate for 30 min, while shaking or rotating in tubes. Ensure that the beads remain in solution.
7. Wash beads three times with 1 ml of water.
8. Wash beads three times with 1 ml 80% ACN, 0.1% TFA.
9. Resuspend in 1 ml 80% ACN, 0.1% TFA.
10. Beads are ready to use.

4.2.4 Fe^{3+}-IMAC plate preparation for R2-P2

1. Resuspend dried peptides from R2-P1 directly in the plate with 150 µl 80% ACN, 0.1% TFA by shaking and incubating in a bath sonicator for 10 min.
2. Make sure that peptides go completely into solution and there is no precipitate. Insoluble precipitates need to be removed by centrifugation.
3. Prepare an empty plate containing the tip comb.
4. Prepare one plate with the calculated amount of IMAC beads. If the volume is < 50 µl, fill up to 50 µl with 80% ACN, 0.1% TFA.
5. Prepare three wash plates with 150 µl 80% ACN, 0.1% TFA per well.
6. Start the R2-P2 Kingfisher program and follow the instructions on the robot. Leave elution plate position empty and start the protocol. The elution plate is prepared and loaded later in order to prevent evaporation of the NH_4OH, which could compromise elution efficiency.
7. The R2-P2 Kingfisher program will pause after approximately 35 minutes. At this point pipette 50 µl of 50% ACN, 2.5% NH_4OH into each well of the elution plate.
8. Follow robot instructions, load the elution plate and resume the program.

9. Remove all plates after the program finishes.
10. Immediately neutralize the elution by adding 30 µl 75% ACN, 10% formic acid to the plate.
11. See optional step 12 or transfer to a MS vial or MS sample plate.
12. (Optional step that we recommended when processing many samples in parallel): To avoid any carryover of magnetic beads to the LC system, filter samples through a C8 stage tip or stage tip plate. For this, pack a stage tip with one layer of C8 material and perform the following steps:
    a. Condition with 30 µl MeOH.
    b. Wash with 30 µl ACN.
    c. Wash with 30 µl 70% ACN, 0.25% acetic acid.
    d. Hold stage tip on top of MS sample vial.
    e. Adjust sample to 50% ACN and pass sample through stage tip, collecting eluate in the MS vial.
    f. Elute with 30 µl 70% ACN, 0.25% acetic acid, collecting this second eluate also in the MS vial.
13. Dry samples down in a speedvac. Dried samples can be store at -20 ºC. Resuspend in 4% formic acid and 3% ACN prior to LC-MS analysis.

4.2.5 R2-P2 KingFisher™ Flex protocol

Protocol report
R2-P2_Fe-IMAC
4/18/2019 2:04:29 PM-07:00 1/4

General info

Protocol information

Protocol name	R2-P2_Fe-IMAC
Modified by	admin
Kit name	R2-P2_Fe-IMAC
Description	Fe3+ IMAC KingFisher Flex 96 protocol for enrichment of phosphopeptides from complex biological samples<br>Mario Leutert, Villen Laboratory, University of Washington

Reagent info

Beads		KingFisher 96 KF plate	
Name	Well volume [µl]	Total reagent volume [µl]	Type
Fe-IMAC beads in 80%ACN, 0.1%TFA	150	-	Reagent

Peptides		KingFisher 96 KF plate	
Name	Well volume [µl]	Total reagent volume [µl]	Type
Sample (peptides) in 80%ACN, 0.1%TFA	150	-	Sample

Wash 1		KingFisher 96 KF plate	
Name	Well volume [µl]	Total reagent volume [µl]	Type
Binding buffer - 80%ACN, 0.1%TFA	150	-	Reagent

Wash 2		KingFisher 96 KF plate	
Name	Well volume [µl]	Total reagent volume [µl]	Type
Binding buffer - 80%ACN, 0.1%TFA	150	-	Reagent

Wash 3		KingFisher 96 KF plate	
Name	Well volume [µl]	Total reagent volume [µl]	Type
Binding buffer - 80%ACN, 0.1%TFA	150	-	Reagent

Elution		KingFisher 96 KF plate	
Name	Well volume [µl]	Total reagent volume [µl]	Type
Elution buffer 50%ACN, 50% 1:20 ammonia	50	-	Reagent

Tip Plate		KingFisher 96 KF plate	
Name	Well volume [µl]	Total reagent volume [µl]	Type
-	-	-	-

Steps data

Tip1		96 DW tip comb	
	Pick-Up	Tip Plate	
	Mix and Collect Beads	Beads	
	Beginning of step	Precollect	No
		Release beads	No
	Mixing / heating:	Mixing time, speed	00:00:15, Bottom mix
		Heating during mixing	No
	End of step	Postmix	No
		Collect count	5
		Collect time [s]	10
	Bind	Peptides	
	Beginning of step	Precollect	No
		Release time, speed	00:01:00, Medium
	Mixing / heating:	Mixing time, speed	00:30:00, Medium
		Heating during mixing	No
	End of step	Postmix	No
		Collect count	5
		Collect time [s]	10
	Wash1	Wash 1	
	Beginning of step	Precollect	No
		Release time, speed	00:01:00, Medium
	Mixing / heating:	Mixing time, speed	00:01:00, Medium
		Heating during mixing	No
	End of step	Postmix	No
		Collect count	5
		Collect time [s]	10
	Wash2	Wash 2	
	Beginning of step	Precollect	No
		Release time, speed	00:01:00, Medium
	Mixing / heating:	Mixing time, speed	00:01:00, Medium
		Heating during mixing	No
	End of step	Postmix	No
		Collect count	5
		Collect time [s]	10
	Wash3	Wash 3	
	Beginning of step	Precollect	No
		Release time, speed	00:01:00, Medium
	Mixing / heating:	Mixing time, speed	00:01:00, Medium
		Heating during mixing	No
	End of step	Postmix	No
		Collect beads	No

Pause - Load Elution Plate		Elution	
		Message	Load Elution plate
		Dispensing volume [µl]	0
Collect Beads		Wash 3	
		Collect count	5
		Collect time [s]	10
Elution of phosphopeptides		Elution	
	Beginning of step	Precollect	No
		Release time, speed	00:00:30, Bottom mix
	Mixing / heating:	Mixing time, speed	00:01:00, Bottom mix
		Heating during mixing	No
	End of step	Postmix	No
		Collect count	5
		Collect time [s]	10
Dispose Beads		Beads	
		Release time, speed	00:00:10, Fast
Leave		Tip Plate	
