# Supplementary material for: R2‐P2 rapid‐robotic phosphoproteomics enables multidimensional cell signaling studies
Source: Mol Syst Biol. 2019 Dec 19;15(12):e9021. doi: 10.15252/msb.20199021 (PMC6920700; doi:10.15252/msb.20199021)
Supplement: Supplementary file 2 — Expanded View Figures PDF [file MSB-15-e9021-s002.pdf]

Expanded View Figures

Figure EV1. Evaluation of protein capture, desalting, digestion, and elution efficiency for R2-P1.

A Binding and washing conditions for R2-P1 were tested on 50 μg yeast lysate. Yeast lysate was brought to pH 2 or pH 8, respectively; and ethanol or acetonitrile was added to reach the indicated dilutions (v/v). Beads were washed three times in the corresponding binding buffer. Proteins were eluted and resolved on a SDS–PAGE.

B 50 μg of yeast protein extract was processed and digested using R2-P1. Elution 1 consisted of 25 mM ammonium bicarbonate with trypsin, elution 2 was water and elution 3 was 1% SDS. The flow through from the magnetic beads binding step was dried and digested in-solution with trypsin. All four samples were additionally desalted using C18 stage tips in this case. 1/50th of each sample was injected on the MS. Shown is a boxplot of the MS1 peptide intensity measured for the different samples (n = 1).

C Total intensity traces for the samples measured in (B).

D Density plots of the distribution of molecular weight, GRAVY score, and isoelectric point of the proteins identified in the R2-P1 processed samples and the flow through of the R2-P1 protein binding step.

E Samples consisting of 50 μg yeast protein extract were prepared by R2-P1 and digested using trypsin or LysC at an enzyme:protein ratio of 1:50, 1:100, 1:200, and 1:400, respectively. Peptide identifications are displayed (mean ± SD, n = 2).

F Fraction of missed-cleaved peptides from (E) (mean ± SD, n = 2).

© 2019 The Authors

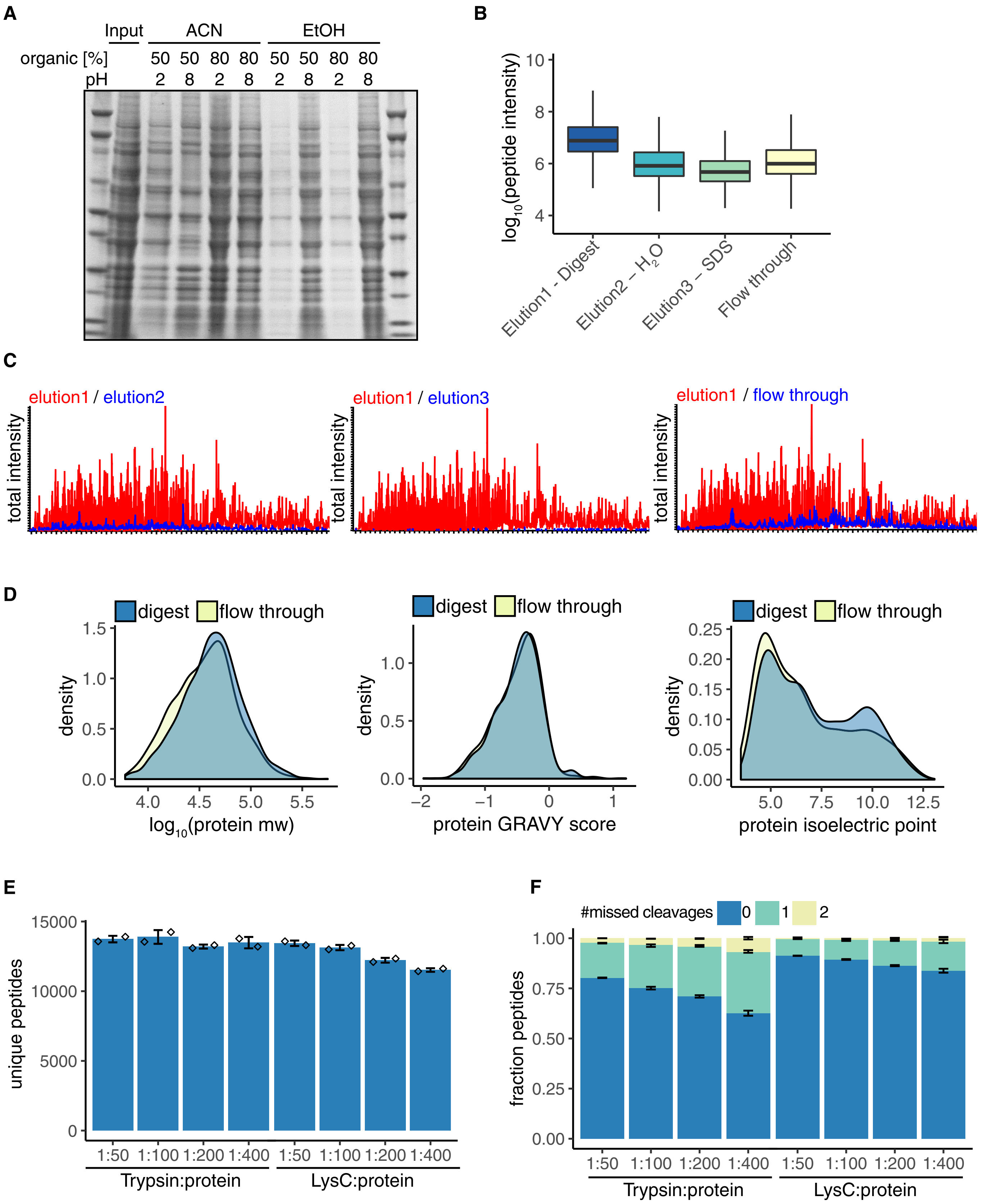

Figure EV1.

 © 2019 The Authors

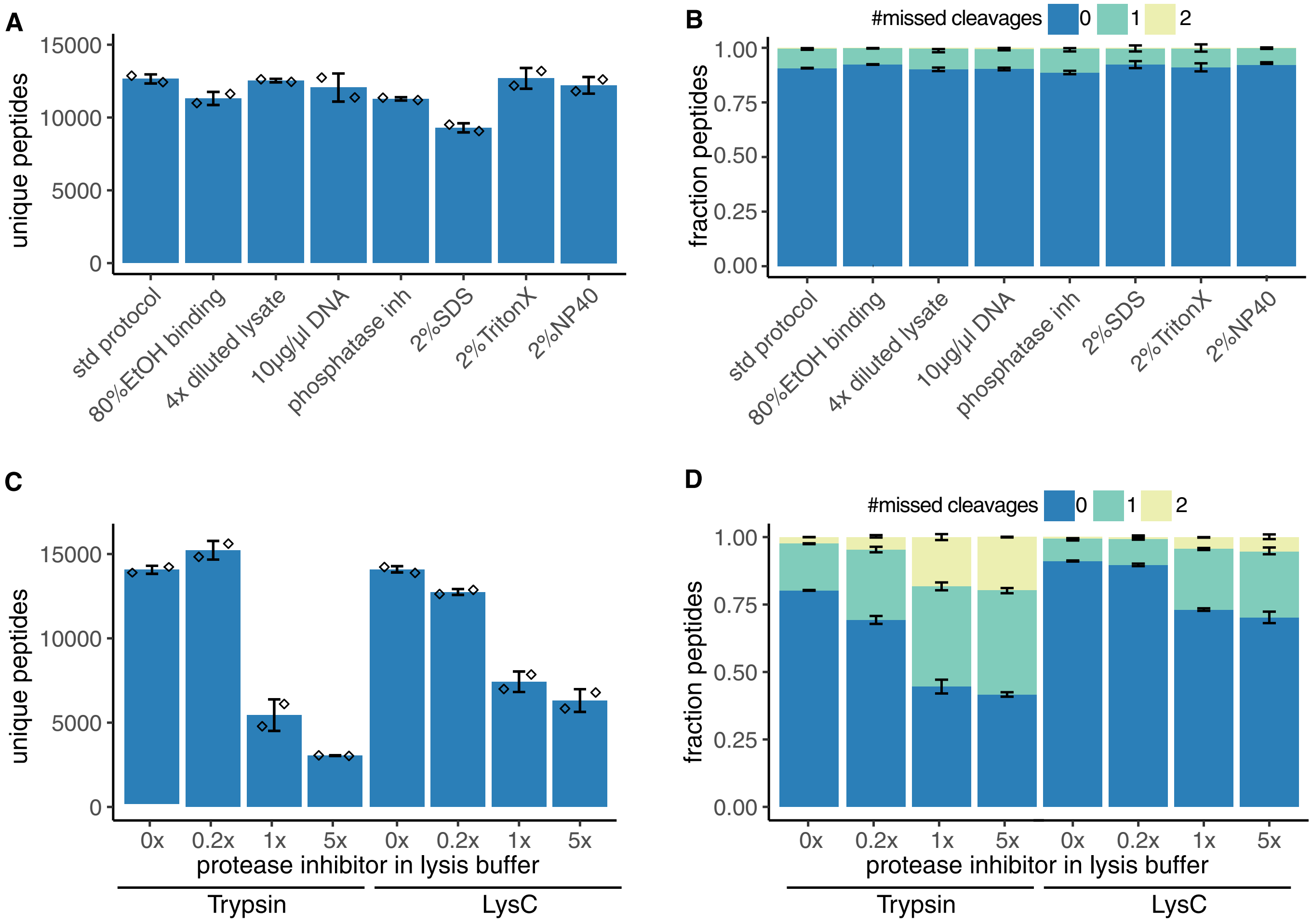

Figure EV2. Evaluation of lysis buffer composition for R2-P1 for total proteome analysis.

200 µg of yeast protein extract in different buffer compositions was processed by R2-P1 followed by peptide analysis with DDA-MS.

A Count of unique peptides is shown for the different conditions (mean ± SD, *n* = 2).
B Fraction of peptides with 0, 1, or 2 missed cleavages identified in (A) (mean ± SD, *n* = 2).
C Different amounts of protease inhibitors were added to 50 µg yeast protein extract to reach 0.2×, 1×, and 5× protease working concentrations relative to the binding and digestion buffer volume. Samples were processed by R2-P1 using either trypsin or lysC for digestion. Peptide identifications are displayed (mean ± SD, *n* = 2).
D Fraction of phosphopeptides with 0, 1, or 2 missed cleavages identified in (C) (mean ± SD, *n* = 2).

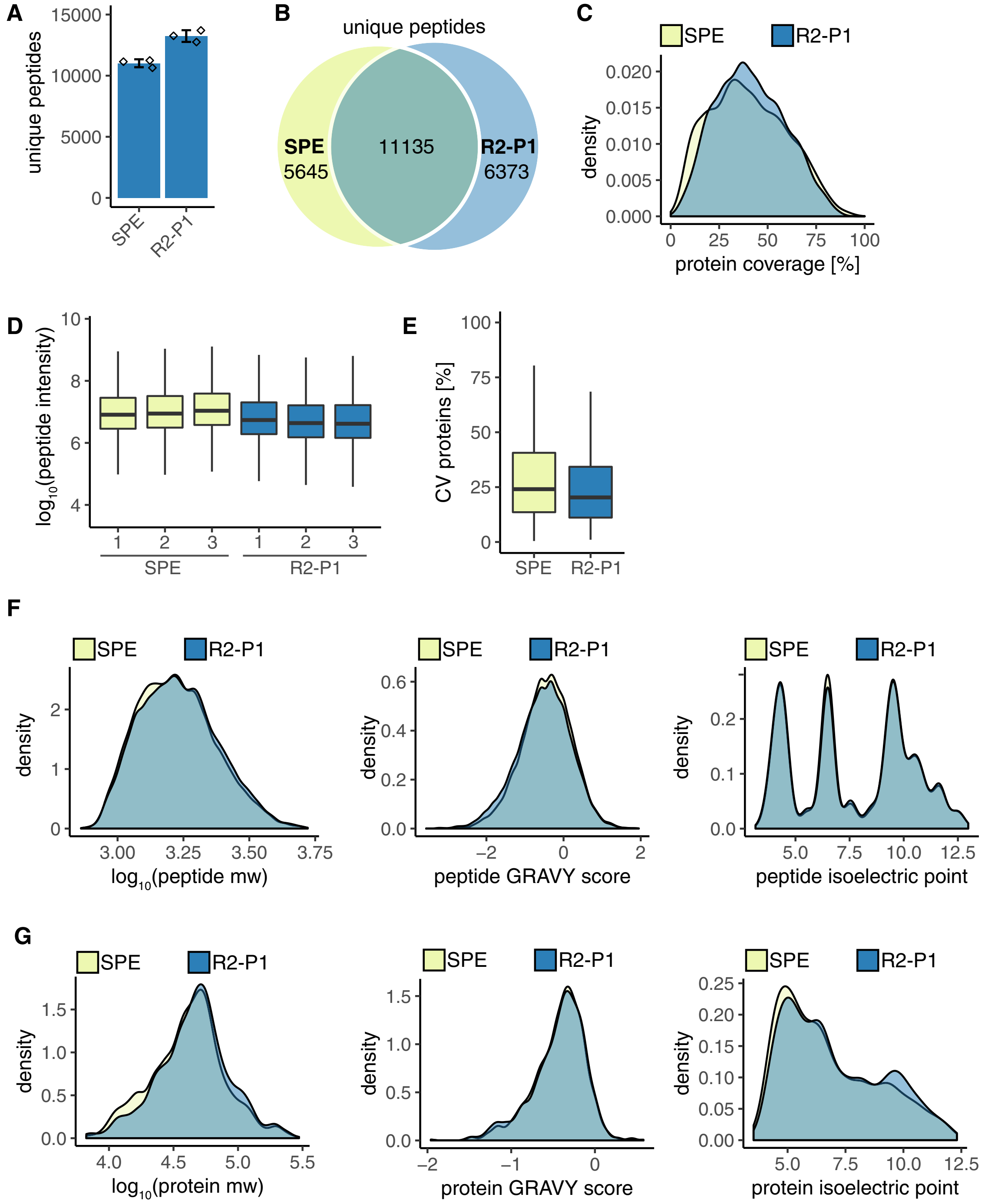

Figure EV3. Comparison of total proteome analysis with R2-P1 versus in-solution digestion C18 SPE desalting.

25 µg of yeast protein extract was processed by R2-P1 or by manual in-solution digestion and reversed-phase C18 SPE desalting in triplicates. Peptide analysis was performed by DDA-MS.

- A Number of unique peptides (mean ± SD, n = 3).
- B Venn diagram of peptides identified in samples processed by SPE or R2-P1.
- C Density plot of protein coverage distribution for the two methods.
- D Boxplot of MS1 peptide intensities (n = 3).
- E CVs of protein intensities for the two sample processing methods (n = 3).
- F Density plots of the distribution of molecular weight, GRAVY score, and isoelectric point of the peptides identified in the two different sample processing methods.
- G Density plots of the distribution of molecular weight, GRAVY score, and isoelectric point of the proteins identified in the two different sample processing methods.

 © 2019 The Authors

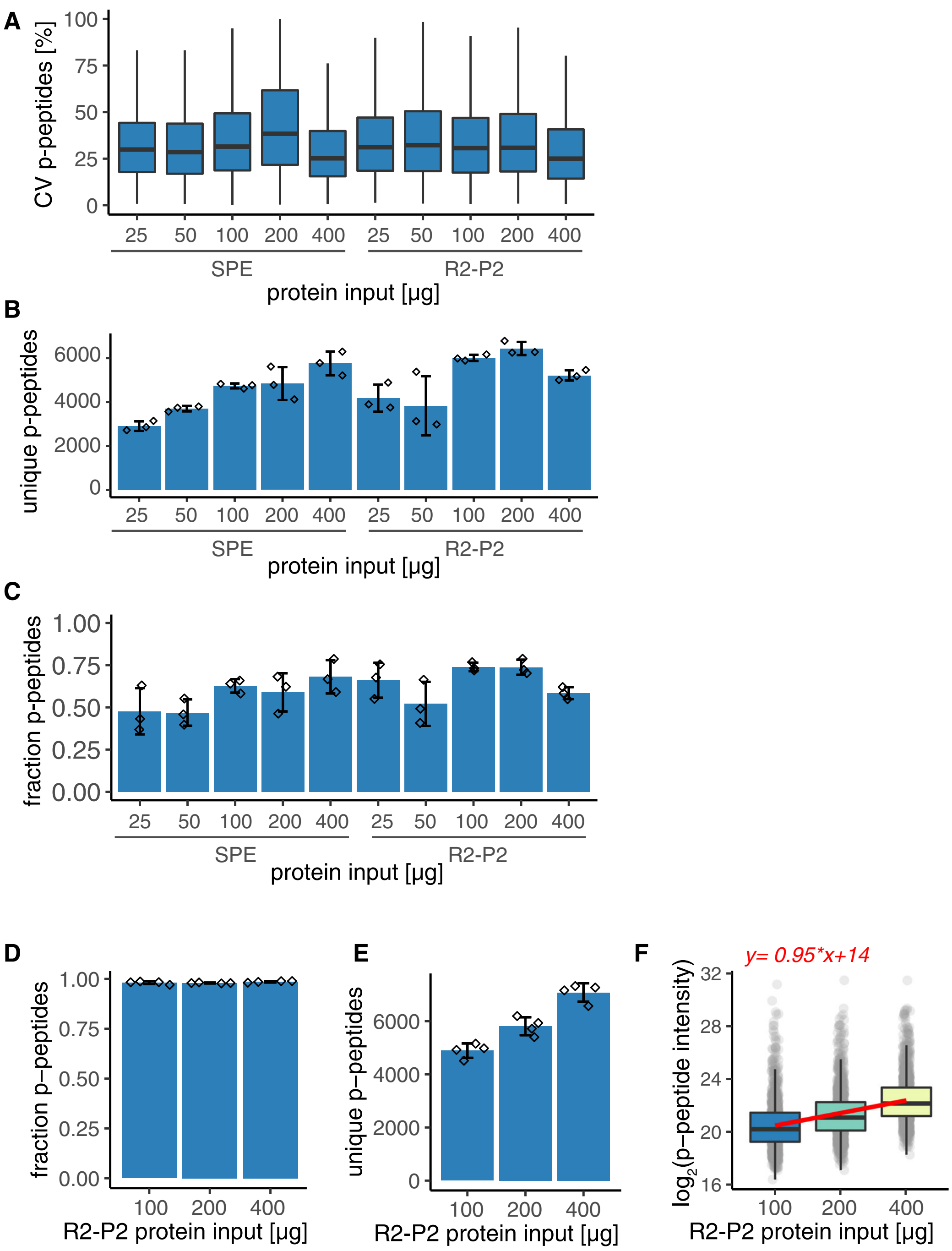

Figure EV4. Comparison and scalability of R2-P2 and SPE.

A Different amounts (25, 50, 100, 200, and 400 µg) of yeast protein extract were processed by R2-P2 or by in-solution digestion and reversed-phase C18 SPE desalting, phospho-enriched by robotic Fe^{3+}-IMAC followed by peptide analysis with DDA-MS. Sample preparation was performed in triplicate. Boxplot depicting CVs for replicate analysis of phosphopeptide MS1 signal intensities (n = 3).

B Number of unique phosphopeptides for samples shown in (A) (mean ± SD, n = 3).

C Fraction of phosphorylated peptides for samples shown in (A) (mean ± SD, n = 3).

D 100, 200, and 400 µg yeast protein extract were processed with R2-P2 followed by peptide analysis with DDA-MS. Sample preparation was performed in quadruplicate. Fraction of phosphorylated peptides (mean ± SD, n = 4).

E Number of unique phosphopeptides for samples shown in (D) (mean ± SD, n = 4).

F Boxplot depicting mean phosphopeptide MS1 signal intensity distributions for samples shown in (D) (n = 4). Red line represents the linear regression of the median phosphopeptide intensities, and the corresponding function is shown on top.

© 2019 The Authors

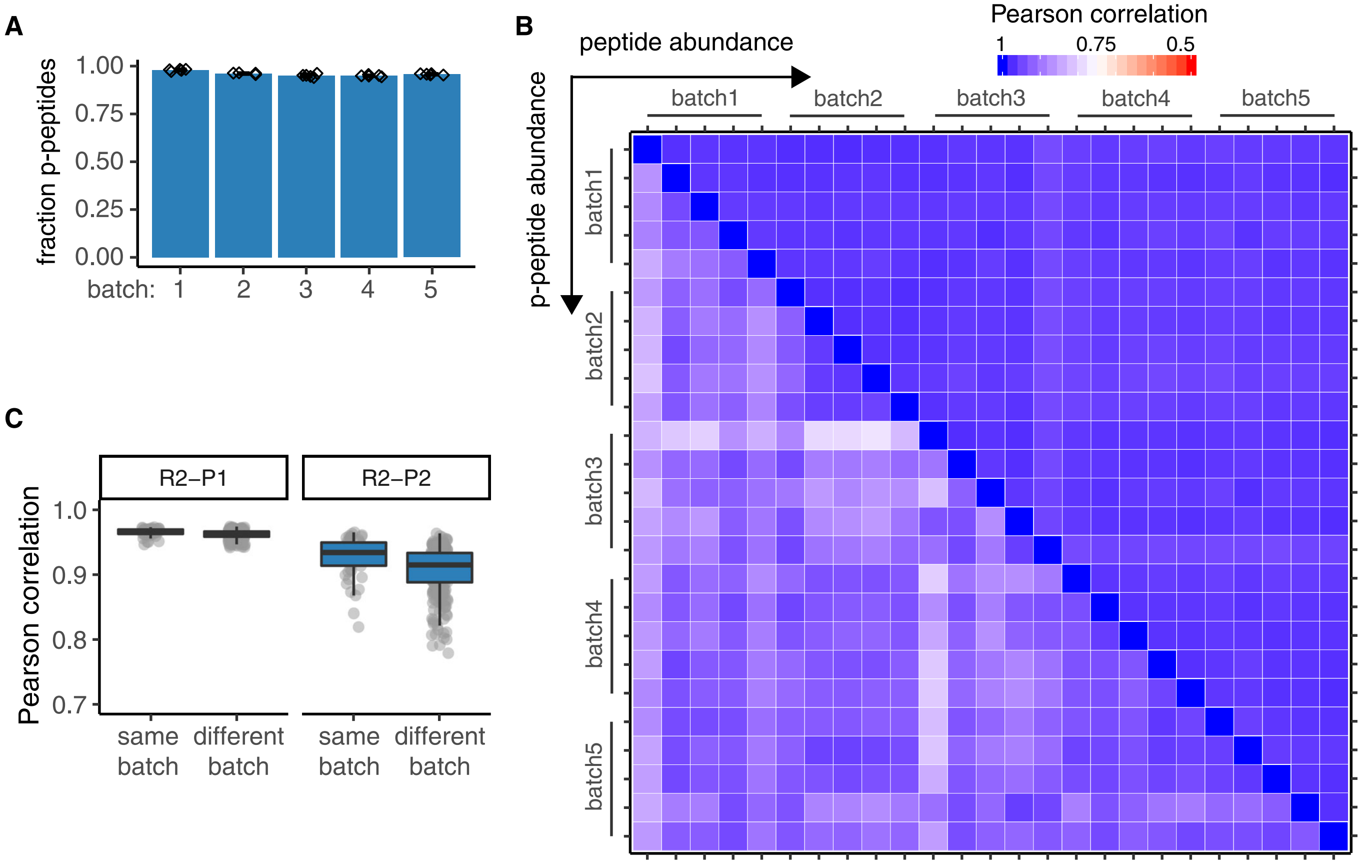

Figure EV5. Reproducibility of R2-P1 and R2-P2.

A Fraction of phosphorylated peptides for R2-P2 batches conducted on five different days (mean ± SD, n = 5).

B Heatmap of Pearson's correlation coefficients of peptide intensities for peptides derived from performing R2-P1 of 25 replicates (five replicates per batch, five batches on five different days) (top right corner) and for phosphopeptides derived from R2-P2 of the same samples (bottom left corner) (n = 25).

C Boxplot depicting distribution of Pearson's correlations for R2-P1 and R2-P2 performed in the same batch on the same day (n = 50) or in batches prepared on different days (n = 250).

 © 2019 The Authors

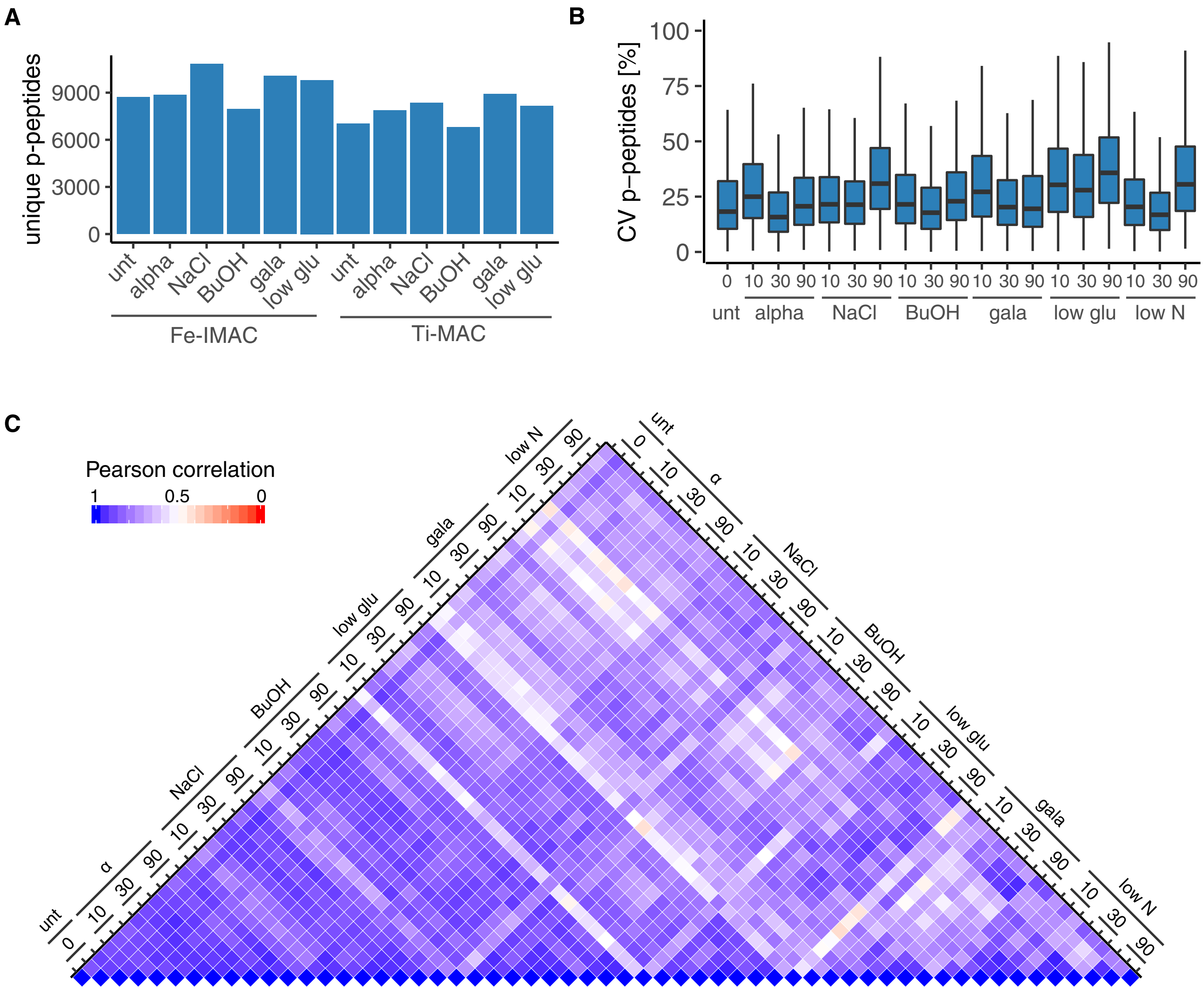

Figure EV6. Library generation and R2-P2 DIA-MS reproducibility.

A Identified phosphopeptides in Fe^{3+}-IMAC and Ti^{4+}-IMAC fractions of pooled time points for the different treatments measured in single-injection DDA-MS experiments.

B CVs for phosphopeptide quantification in the DIA-MS experiment (n = 3).

C Heatmap of Pearson's correlation coefficients for all DIA-MS measurements.

© 2019 The Authors

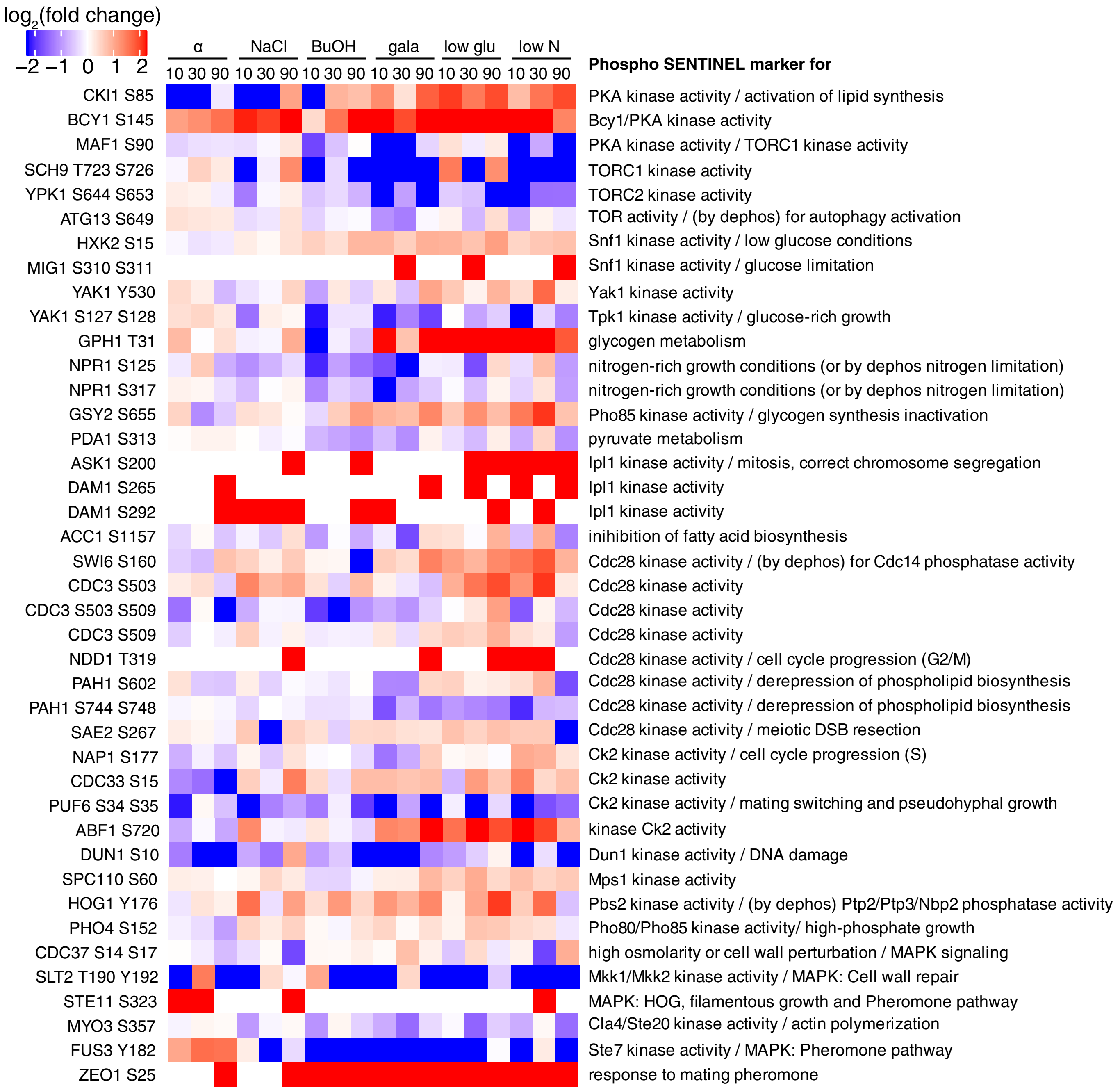

Figure EV7. Phosphosite markers for biological processes.

Phosphosites were mapped to phosphopeptide sentinel markers as defined by Soste *et al* (Soste *et al*, 2014). Log_2 fold changes over untreated are shown for the different treatment and time points. Phosphosite is indicated on the left side of the heatmap and description for upregulation of the phospho marker on the right.

Figure EV8. Dynamics of phosphosites for TORC1-related proteins.

Quantified phosphosites were mapped onto TORC1 and literature-curated TOR targets from Oliveira *et al* (2015). Each heatmap displays log_2 fold intensity changes over untreated of one or multiple co-occurring localized phosphorylation site(s) belonging to the indicated proteins. Heatmap rows correspond to treatments and columns to time points.

 © 2019 The Authors

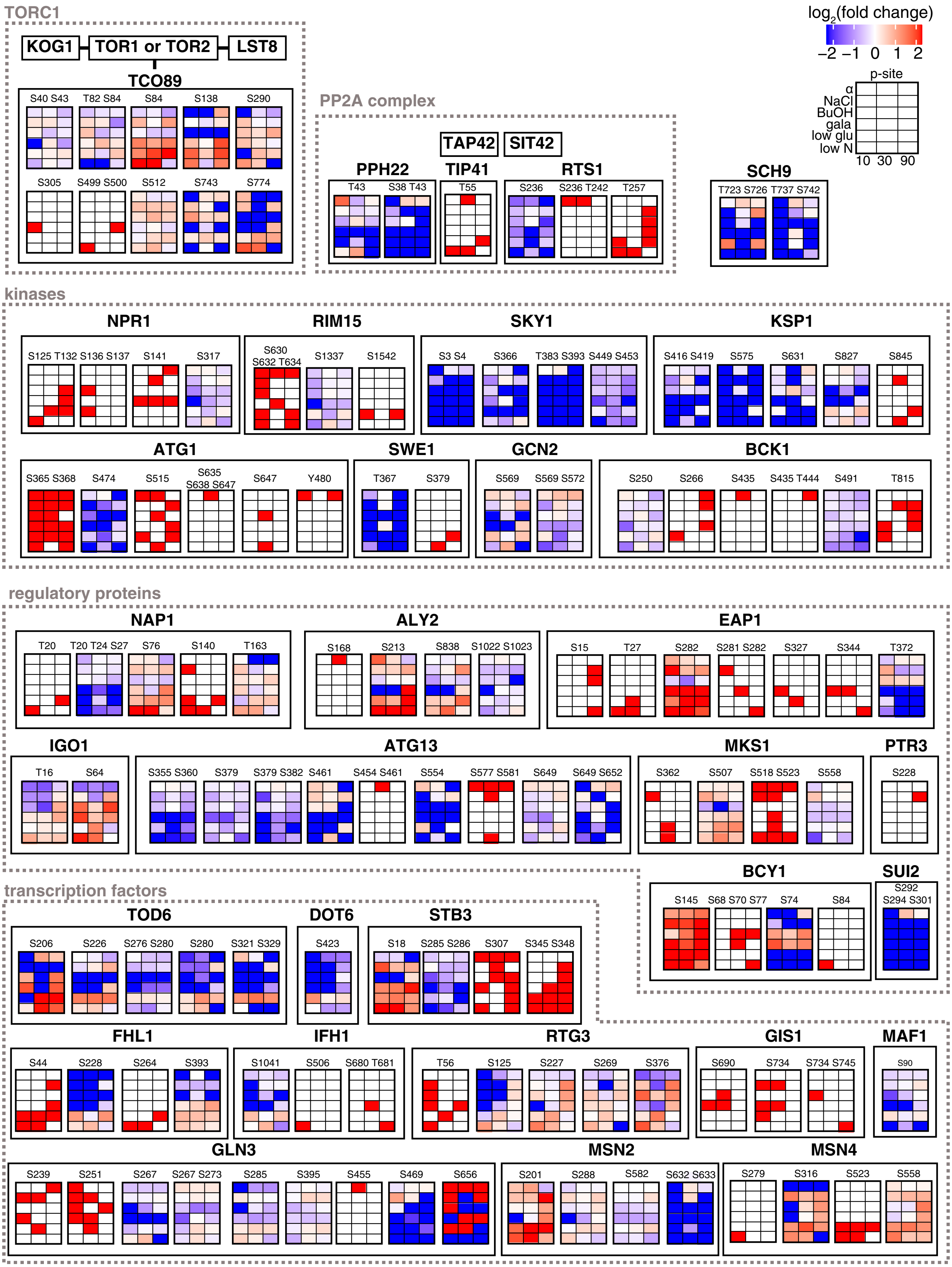
log2(fold change)
−2 −1 0 1 2
p-site
α
NaCl
BuOH
gala
low glu
low N
10 30 90
TORC1
KOG1
TOR1 or TOR2
LST8
TCO89
PP2A complex
TAP42
SIT42
PPH22
TIP41
RTS1
SCH9
kinases
NPR1
RIM15
SKY1
KSP1
ATG1
SWE1
GCN2
BCK1
regulatory proteins
NAP1
ALY2
EAP1
IGO1
ATG13
MKS1
PTR3
BCY1
SUI2
transcription factors
TOD6
DOT6
STB3
FHL1
IFH1
RTG3
GIS1
MAF1
GLN3
MSN2
MSN4

Figure EV8.
